# Response process validity of the 9-item shared Decision-Making Questionnaire (SDM-Q-9) in a cognitive interview study with patients with cancer

**DOI:** 10.1038/s41598-025-99640-2

**Published:** 2025-05-03

**Authors:** Hannah Sigl, Levente Kriston, Isabelle Scholl, Martin Härter, Pola Hahlweg

**Affiliations:** Department of Medical Psychology, University Medical Center Hamburg-Eppendorf, Hamburg, Germany

**Keywords:** Shared decision-making, Response process validity, Construct validity, Patient-reported outcomes, Cancer care, Qualitative interviews, Health care, Health services

## Abstract

The patient-reported, psychometrically evaluated, and frequently used 9-item Shared Decision-Making Questionnaire (SDM-Q-9) assesses shared decision-making processes in medical encounters. This study aimed to investigate SDM-Q-9’s response process validity from the perspective of patients with cancer. We conducted qualitative cognitive interviews with *N* = 11 patients of a German comprehensive cancer center. Participants described their item interpretations, content anchors for highest and lowest scores, and difficulties with item rating. We performed qualitative content analysis and assessed concordance between item interpretations and the underlying theoretical construct of SDM-Q-9. Participants reported it necessary to have a choice (i.e., awareness of more than one option) to answer SDM-Q-9 and had difficulties rating one specific medical encounter only. For most items, interpretations matched the underlying theoretical construct. Discordance was found for item 2 (sharing the decision), and partial discordance for items 5 (supporting comprehension) and 6 (eliciting preferences). Content anchors for highest and lowest ratings showed substantial inter-individual variance. Results regarding SDM-Q-9’s response process validity suggest validity for most items. However, reported prerequisites to be able to answer SDM-Q-9 and discordance between the underlying theoretical construct and interpretations found for three items warrant further investigation, before revision of the measure should be considered.

**Supplementary Information:**

The online version contains supplementary material available at 10.1038/s41598-025-99640-2.

## Introduction

Shared decision-making (SDM) has been argued to be “the pinnacle of patient-centered care”^1^ and is a central component of current high-quality healthcare. SDM presumes at least two parties, usually physician and patient, to be involved in the decision-making process by exchanging information and preferences about the available treatment options^2^. They jointly balance benefits and risks as well as possible consequences of different treatment options (including no treatment) and, as a result, come to a treatment decision that both parties can agree on^2,3^. Clinical situations with equipoise (i.e., uncertainty among medical experts about the best choice out of more than one available options) and substantial consequences of the available options on the patient’s subsequent quality of life have been argued to make SDM particularly beneficial^4,5^. Thus, SDM is important in cancer care^6,7^.

In order to effectively assess and implement SDM, it is important to be able to accurately measure it. SDM can be measured from different perspectives: (1) patient-reported measures, e.g., 9-item Shared Decision Making Questionnaire (SDM-Q-9)^8^ or collaboRATE™ measure^9^; (2) physician-reported measures, e.g., physician version of the Shared Decision Making Questionnaire (SDM-Q-Doc)^10^; and (3) observer ratings, e.g., Observer OPTION™ scale^11^. Over the last decades, many new SDM measures have been developed^12^. Most of them are patient-reported and the majority showed good reliability^13^. However, the evaluation of validity was found to be largely lacking^13,14^.

The SDM-Q-9, developed by authors of this publication (MH, IS, LK) and colleagues, is one of the most frequently used patient-reported instruments for measuring SDM in medical encounters^8,15,16^. It consists of two open-ended introductory questions on the health problem and the decision made as well as nine items (see Table 1 for item wording). The items are scored on a 6-point Likert-scale ranging from “completely disagree” to “completely agree”. Nine steps of the SDM process served as a theoretical basis for the development of the SDM-Q-9^8,17,18^ (see Table 1). These were based on Elwyn’s model of competencies for involving patients^19^ and theories from general psychology, social psychology, and decision analysis^17^. As of August 2024, the SDM-Q-9 is available in 24 languages; further translations are in progress (see www.sdmq9.org). Regarding psychometric properties, high internal consistency (Cronbach’s α > 0.9), high item discrimination, and a response rate over 80% were found, indicating good reliability and acceptance^8^. First results showed high face and factorial validity^8^. However, convergent validity between the SDM-Q-9 and the Observer OPTION scale could not be established^16^. To our knowledge, response process validity of the SDM-Q-9 has not yet been investigated with sound methodology from the patients’ perspective^8,20–25^, as required in the consensus-based standards for the selection of health measurement instruments (COSMIN) criteria^26^. Response processes are “an opportunity for us to understand the meaning of scores from psychological measures”^27^. We define response process validity as “the thought processes of survey participants aligning with the underlying construct and conceptualization intended by the survey developers and researchers”^28^. While assessing response process validity was less common when the SDM-Q-9 was originally developed, the necessity to thoroughly evaluate response process validity of the questionnaire, one central aspect of construct validity^28^, became apparent when it became widely used in routine care and in implementation studies.

**Table 1:** SDM-Q-9 item labels, items, and the underlying theoretical construct.

Item Nr.	Label ^a^	SDM-Q-9 item	Step of SDM process according to the underlying theoretical construct
1	Focusing the decision	My doctor made clear that a decision needs to be made.	Disclosure that a decision needs to be made
2	Sharing the decision	My doctor wanted to know exactly how I want to be involved in making the decision.	Formulation of equality of partners
3	Presenting options	My doctor told me that there are different options for treating my medical condition.	Presentation of treatment options
4	Informing on options	My doctor precisely explained the advantages and disadvantages of the treatment options.	Informing on the benefits and risks of the options
5	Supporting comprehension	My doctor helped me understand all the information.	Investigation of patient’s understanding and expectations
6	Eliciting preferences	My doctor asked me which treatment option I prefer.	Identification of both parties’ preferences
7	Deliberating the decision	My doctor and I thoroughly weighed the different treatment options.	Negotiation
8	Selecting an option	My doctor and I selected a treatment option together.	Reaching a shared decision
9	Planning actions	My doctor and I reached an agreement on how to proceed.	Arrangement of follow-up

^a^These labels were proposed by Kriston et al.^43^ DOI: 10.1111/hex.13130.

Thus, the aim of this study was to investigate the response process validity of SDM-Q-9, a well-established and frequently used patient-reported SDM questionnaire, by assessing the validity of the response process in patients with cancer and evaluating the participants’ item interpretations.

## Methods

### Study design

A qualitative cross-sectional study was conducted to gain insights into the response process validity of SDM-Q-9 analyzing data from cognitive interviews with patients with cancer. Cognitive interviewing pays “explicit attention to the mental processes respondents use to answer survey questions and thus allows covert as well as overt problems to be identified”^29^. It is an approved and suitable approach to gain a deeper understanding of the test takers thought processes while answering the items of the questionnaire^29,30^. We followed the consolidated criteria for reporting qualitative research checklist (COREQ)^31^ and the COSMIN reporting guideline for studies on measurement properties of patient-reported outcome measures^32^, where applicable (cp. Additional files 1 and 2).

### Setting and subjects

SDM-Q-9 was used as the primary outcome in a large-scale cluster-randomized SDM implementation trial in cancer care^33,34^. As an additional quality improvement strategy, we decided to evaluate the response process validity of the measure. Hence, we analyzed data from a sample of patients with cancer. Participants were outpatients of the University Cancer Center Hamburg (UCCH), a substructure of the University Medical Center Hamburg-Eppendorf (UKE). Participants had to be 18 years or older, had to have a self-reported cancer diagnosis, and had to have had a clinical encounter regarding their cancer disease not more than two weeks prior to the interview. People with insufficient language skills in German or severe cognitive impairment that made participation in cognitive interviews impossible were excluded.

### Data collection

A member of the study team (HS) approached potential participants at the oncological outpatient clinic’s waiting areas at UKE (convenience sampling). They were informed about the study and its aims verbally and in written form, and gave written informed consent prior to participation. The interview appointments were scheduled for another day at the Department of Medical Psychology of the UKE. According to the COSMIN criteria, we planned to conduct at least seven interviews^26^. Participants were compensated for their participation with 25 Euros. Cognitive interviews were conducted between December 2019 and January 2020 by HS and followed an interview guide (see Additional file 3). HS was a female master’s student in clinical psychology and psychotherapy at the time of the interviews and was supervised by PH, a clinical psychologist experienced in conducting qualitative interviews with patients with cancer.

One-time cognitive interviews were conducted in person at the Department of Medical Psychology at the University Medical Center Hamburg-Eppendorf. During the cognitive interviews, only the participant and the interviewer were present. Participants were first asked to fill out the SDM-Q-9 as a paper-and-pencil version while applying the think-aloud technique (i.e., participants verbalized their thoughts while completing the questionnaire)^29,30^. Then, the probing technique was used (i.e., the interviewer asked follow-up questions to clarify participants’ thought process)^29,30^. Participants were asked to share their individual interpretation of each item, to describe content anchors for highest and lowest scores, and to report any difficulties.

Additionally, participants completed a short survey at the end of each interview to describe the sample. This survey included demographic questions, the Control Preferences Scale (CPS)^35^, as well as the German 16-item version of the European Health Literacy Questionnaire (HLS-EU-Q16)^36^. The CPS is a widely used instrument to assess the preferred extent of patients’ participation in healthcare decision-making (ranging from physician-led through shared to patient-led decision-making) with one question^35^. The HLS-EU-Q16 is the short version of the HLS-EU-Q, a generic (i.e., not disease specific) measurement tool developed by the European Health Literacy consortium to assess health literacy across Europe^37^. Health literacy means one’s ability to access, understand, and use health information^37^. In the HLS-EU-Q16, health literacy sum scores with a range from 1 to 16 are divided into three categories: “inadequate” for sum scores of 1 to 8, “problematic” for sum scores of 9 to 12, and “sufficient” for sum scores of 13 to 16^36^.

### Data analysis

Interviews were audio-recorded, transcribed verbatim, and anonymized. Transcripts were not returned to participants. HC made notes during the interview to structure the interview process. These were not included in data analysis. Qualitative content analysis with an inductive approach was performed to analyze the data, following the approach by Mayring and colleagues^38,39^. This approach has been defined “as an approach of empirical, methodological controlled analysis of texts within their context of communication, following content analytical rules and step by step models, without rash quantification”^38^ and is clearly structured with “strict category relatedness and rule orientation”^40^. It is appropriate, if one wants to focus on codes emerging from the content of the material rather than linguistics or more interpretative analysis such as hermeneutic approaches^40^. The following aspects were analyzed for each item: (a) prerequisites for answering the item (i.e., conditions that need to be met for the item and its rating to make sense); (b) interpretations of the item; (c) content anchors for highest and lowest ratings (i.e., certain physician behavior or circumstances that would lead to the participant rating highest or lowest scores); and (d) difficulties with the item. Furthermore, general prerequisites for and difficulties with the SDM-Q-9 were assessed. Coded text passages could be as short as a meaningful part of a sentence and as long as the entire text section on one topic. Multiple codings with the same code within one transcript were allowed. In the analysis we prioritized inclusivity by reporting all aspects voiced by participants (i.e., also aspects voiced by one person only). This allowed us to catch also those aspects experienced by few. Data analysis consisted of five steps. Transcripts were coded by one researcher (HS), with continuous supervision and review by a second researcher (PH). First, the main coder (HS) read the entire data set to gain an overview and then coded about 50% of the material (i.e., half the transcripts). The coder coded the transcripts, but would have had the option to go back to the audio recordings if transcripts would have been unclear. Second, the second researcher (PH) gave feedback on the preliminary coding scheme (including codes, code descriptions, and partial codings), followed by discussion (HS and PH). Coding scheme and all prior codings were revised accordingly. Third, the remaining 50% of the material were coded by HS. Where necessary, additional categories were created and integrated. Fourth, the coding scheme was again discussed within the team (PH, HS), and coding scheme and codings were revised accordingly (HS). Fifth, the senior researcher (PH) carefully reviewed and revised the coding scheme and codings one more time. Throughout the coding process, disagreements were resolved by discussion in the research team until consensus was reached. Participants did not give feedback on the findings. Additionally, descriptive statistics were calculated for demographic data. For transcription the software f4 (version 4, dr. dressing & pehl GmbH, Marburg, Germany), for qualitative analyses MAXQDA software (version 12, VERBI GmbH, Berlin, Germany), and for descriptive statistics SPSS (version 25, IBM Corp., Armonk, NY) were used.

### Ethics approval and consent to participate

The study was approved by the Local Psychological Ethics Committee of the UKE (LPEK-0081) and followed principles of good scientific practice as stated by the Declaration of Helsinki of the World Medical Association. Informed consent was obtained from all individual participants included in the study.

## Results

### Sample characteristics

Sixteen patients with cancer initially agreed to participate. Five of these dropped out before the interview. Reasons were incapacity due to worsened health status (*n* = 2), missed interview due to scheduling issues (*n* = 1), no reason (*n* = 2). Thus, eleven cognitive interviews with patients with cancer were conducted. Of these, four had to be re-scheduled at least once. Interviews lasted between 40 and 76 min (mean = 59, SD = 12.7). Most of the participants were male (72.7%). Age ranged from 32 to 80 years (mean = 53.45, SD = 13.7). The majority of the participants (54.5%) demonstrated “problematic” health literacy and two participants (18.2%) displayed “inadequate” health literacy according to widely used thresholds of the HLS-EU-Q16. For additional sample characteristics, see Table 2.

**Table 2:** Sample characteristics (*N* = 11 patients with cancer).

Sample characteristic		
	*Mean*	*SD*
Age in years	53.5	(13.7)
	*n*	*%*
Gender		
Female	3	(27.3)
Male	8	(72.7)
Mother tongue		
German	9	(81.8)
Other	2	(18.2)
Formal education		
Low^a^	2	(18.2)
Intermediate^b^	1	(9.1)
High^c^	3	(27.3)
Very high^d^	5	(45.5)
Occupational status		
(Self-)employed	9	(81.8)
Retired	2	(18.2)
Time since initial diagnosis		
< 1 year	3	(27.3)
1–5 years	3	(27.3)
> 5 years	3	(27.3)
Missing	2	(18.2)
Current status of cancer disease		
Locally limited	3	(27.3)
Metastasized	5	(45.5)
In remission	2	(18.2)
No information	1	(9.1)
CPS: “Who do you think should make the treatment decision?”		
Only myself	0	(0.0)
Mostly myself	2	(18.2)
Me and my doctor	8	(72.7)
Mostly my doctor	1	(9.1)
Only my doctor	0	(0.0)
HLS-EU-Q16		
“Sufficient” health literacy^e^	3	(27.3)
“Problematic” health literacy^f^	6	(54.5)
“Inadequate” health literacy^g^	2	(18.2)

N = number; sd = standard deviation; cps = control preferences scale; HLS-EU-Q16 = 16-item version of the European health literacy questionnaire (sum scores, range 1–16); ^a^Low = no formal degree or graduation after less than 10 years at school; ^b^Intermediate = graduation after 10 or 11 years at school; ^c^High = graduation after more than 11 years at school; ^d^Very high = college or university degree; ^e^HLS-EU-Q16 sum score of 13–16; ^f^HLS-EU-Q16 sum score of 9–12; ^g^HLS-EU-Q16 sum score of 1–8.

### Prerequisites and general difficulties for rating the SDM-Q-9

The full coding scheme including codes, memos, frequencies of codings, frequencies of coded documents, and exemplary quotes for each code can be found in Additional file 4. The vast majority of participants noted the existence of more than one suitable treatment option as a prerequisite for being able to fill out the SDM-Q-9. This prerequisite was mentioned for the SDM-Q-9 in general as well as most of the individual items. Some participants reported there had been only one suitable treatment option in their case. In addition, some participants voiced that a decision against a possible treatment in the face of a serious illness was not an option to them, despite its theoretical availability (see item 6, eliciting preferences, Additional file 4). As a result, these participants had difficulties to answer the SDM-Q-9. Furthermore, for items 4 (informing on options) and 5 (supporting comprehension), some participants reported that patients need to be sufficiently receptive (i.e., not overwhelmed by emotions or otherwise preoccupied). Only then the physician can explain benefits and risks and enable understanding, and the patient can rate items 4 and 5. For items 8 (selecting an option), one participant each voiced a trustful patient-physician-relationship and the patient wanting to be involved as additional prerequisites. Item 9 (planning actions) was the only item without prerequisites. If prerequisites were not met, participants experienced difficulties rating the SDM-Q-9.

Several participants reported difficulties rating one specific clinical encounter only. They tended to combine multiple experiences they have had with the respective physician. Furthermore, some participants criticized that the German term for SDM (“Partizipative Entscheidungsfindung”) was hard to understand, that they felt the need for further explanations beyond checking a box on the questionnaire, and that the SDM-Q-9 was not cancer-specific. Additionally, some patients perceived strong similarities and interdependency between item 6, 7, and 8. The identification of preferences (item 6) and the weighing of options (item 7) were reported to be hardly separable. Also, reaching a decision (item 8) was described as a direct consequence of weighing options (item 7). Furthermore, for items 4 (informing on options) and 5 (supporting comprehension), participants talked about the need to receive complete information (cp. Additional file 4). However, it remained unclear how patients can know whether they received complete information.

### Item interpretations and congruence with the underlying theoretical construct

We elaborate on major findings in the following. Further details per item on patient interpretations of the items, their content anchors for situations, behaviors leading to highest and lowest scores, and potential difficulties for answering each item are described in Additional file 4.

The comparison between the participants’ item interpretations and the underlying theoretical construct (see Table 3) indicated that the patient interpretations of most items matched the theoretical construct (items 1, 3, 4, 7, 8, and 9). However, differences between participants` interpretations and the underlying theoretical construct were found for item 2 (sharing the decision), and to a lesser extent for items 5 (supporting comprehension) and 6 (eliciting preferences).

For item 2 (sharing the decision), participants rather linked the actual opportunity to be involved in the decision-making process to the rating of this item than the explicit designation of the equality of partners by the physician. Several participants did not perceive it necessary to be explicitly asked how they wanted to be involved. In their opinion, this unnecessity might be why the physician failed to do so. As a result, participants had difficulties to rate the item. Item 2 is the item that was found to be most problematic regarding congruence between the underlying theoretical construct and participants’ understanding of the item.

For items 5 (supporting comprehension) and 6 (eliciting preferences), partial congruence between patient interpretations and the theoretical construct were shown. However, this was most likely due to a lack of correspondence between the item wording and the content of the underlying theoretical construct. For item 5, the underlying theoretical construct calls for the investigation of the patient’s understanding and expectations. However, the actual wording of the item only asks for patient understanding. Thus, the partial congruence was due to the item wording and not the patients’ interpretation. For item 6, the item wording focuses on the patient’s preference, while the theoretical construct includes both parties’ preferences.

**Table 3:** Comparison of the patient-reported item interpretations with the underlying theoretical construct of the SDM-Q-9 (*N* = 11 patients with cancer).

Item nr.	SDM-Q-9 item	Patient-reported item interpretation(s)	Congruence with underlying theoretical construct (Yes/No/Partial)
1	My doctor made clear that a decision needs to be made	The physician made clear that the need for making a decision is given	Yes
2	My doctor wanted to know exactly how I want to be involved in making the decision	The patient had the opportunity for involvement in the decision-making process (independently of if it happened or not) The physician explained diagnostic findings and asked for the patient’s perspective	No (Participants did not require explicit discussion regarding equality)
3	My doctor told me that there are different options for treating my medical condition.	The physician presented and explained different treatment options	Yes
4	My doctor precisely explained the advantages and disadvantages of the treatment options	The physician explained the advantages and disadvantages of the treatment options	Yes
5	My doctor helped me understand all the information	The physician explained the information in a comprehensible way using simple language. The physician checked if the patient understood the information The physician answered to questions.	Partial (Participants did not require the investigation of the patient’s expectations, which is also not included in the wording of the item)
6	My doctor asked me which treatment option I prefer	The physician asked the patient about his/her treatment preference The patient weighed the different treatment options.	Partial (Participants did not require the identification of the physician’s preferences which is also not included in the wording of the item)
7	My doctor and I thoroughly weighed the different treatment options	The physician and the patient jointly weighed the different treatment options based on the patient´s individual circumstances	Yes
8	My doctor and I selected a treatment option together.	The physician and the patient decided together.	Yes
9	My doctor and I reached an agreement on how to proceed	The physician and the patient decided on the next steps and/or the further course of treatment	Yes

### Content anchors for highest and lowest ratings

Content anchors for highest ratings were often closely linked to the patient-reported item interpretations (see Additional file 4). Content anchors for lowest scores were frequently linked to non-occurrence of behavior expected for the highest rating or non-compliance with perceived prerequisites. However, sometimes the same content anchor given by one participant for the highest rating was given by another participant for the lowest rating (e.g. item 1, focusing the decision, see Additional file 4). Additionally, for some items it remained unclear if non-compliance with prerequisites would lead to highest or lowest ratings of the item. Some patients reasoned that they would indicate the lowest score because to them the question was not applicable (e.g., item 3, presenting options, see Additional file 4). Others would choose the highest score by hypothesizing how it could have been if the rated clinical encounter would have corresponded to the prerequisites (e.g., item 6, eliciting preferences, see Additional file 4).

## Discussion

To our knowledge, this was the first study to explore the response process validity of the SDM-Q-9 from the patients´ perspective. Our qualitative analysis of cognitive interviews with 11 patients with cancer found the awareness of more than one available treatment option (i.e., equipoise) to be an important prerequisite for being able to rate the SDM-Q-9. If this or other prerequisites were not met, participants reported difficulties choosing the appropriate score. Participants’ interpretations of the items were found to correspond to the underlying theoretical construct for most items. However, a mismatch was seen for item 2, and the wording of items 5 and 6 was found to be only partially congruent with the underlying theoretical construct.

There is a lack of evaluation regarding response process validity of SDM measures both in general and for the SDM-Q-9^13,14^. Thus, this assessment of the SDM-Q-9’s response process validity from the perspective of patients with cancer helped to gain important first insights into the patients’ thought processes while answering the SDM-Q-9, and generated preliminary hypotheses on difficulties and misinterpretations that should be considered when administering the SDM-Q-9.

Almost all participants reported prerequisites that need to be met to be able to rate the SDM-Q-9 with validity. The most important prerequisite was patient awareness of equipoise. This corresponds to theoretical conceptualizations of SDM that define equipoise as a prerequisite for SDM^2,19,41^. The two introductory open-ended questions of the SDM-Q-9 can be interpreted as an attempt to gain insights on the context of the decision discussed, however they do not necessarily tell if more than one treatment option was available. To address this finding, possible avenues that could be tested in future studies include: a thorough pre-selection of clinical situations, further introductory information, an additional item asking whether the patient was aware of more than one treatment option, and/or the option to choose “does not apply: no options/choice”.

Patients with cancer in this study often reported that only one treatment option had been available. Within this study, it was not possible to evaluate whether there was only one evidence-based option available (actual lack of equipoise) or whether the patient was aware of only one treatment option despite more than one being available (perceived lack of equipoise). At least the choice for or against one option is always available. However, choice awareness might be limited due to the physician not communicating it or the patient not recalling it, and some patients voiced that deciding against a possible treatment was not an option to them. Linking SDM-Q-9 ratings on the item level to recordings of clinical encounters could shed light on the open question if the doctor actually presented more than one option to the patient.

If they were not aware of more than one treatment option (i.e., this prerequisite not met), some participants assessed the item as not applicable, which led them to indicate the lowest score. However, other participants rated the highest score because they hypothesized how the situation could have been if the prerequisite would have been met. One could say, they gave the physician the benefit of the doubt. These findings showed that SDM-Q-9 ratings might be exposed to misinterpretations, if the prerequisite of equipoise was not met. Further evaluations of the measure could investigate if adding “not applicable” as a response option would strengthen the SDM-Q-9’s validity. However, consequences for the currently used sum score scoring would have to be considered.

Furthermore, some participants seem to have rated several encounters with the respective physician rather than one specific encounter. This aligns with SDM being conceptualized as a multi-step longitudinal process and as deferring the decision is explicitly mentioned as part of an SDM process^42^. However, this complicates SDM measurement and is currently not sufficiently taken into account in existing measures. We recommend further research investigating different introductory information and on patients focusing on one specific versus more than one encounter.

On the item level, most items seem to be unproblematic. This study generated hypotheses, why difficulties with some items might occur. For item 2 (sharing the decision), participants did not deem it necessary for their physician to explicitly talk about how patients wanted to be involved, which seems to lead to misunderstandings in the item interpretation and rating. Problems with the formulation of the equality of partners were already found in the SDM-Q, the precursory measure to the SDM-Q-9^8^. Additionally, a recently proposed skills network approach to interpret the SDM-Q-9 in a new manner found item 2 (and item 1) to be only moderately connected to other items^43^. If further evaluation of item 2 would replicate the problems found in the study at hand, revision or deletion of item 2 should be considered. The perceived similarity between items 6 (eliciting preferences), 7 (deliberating the decision), and 8 (selecting an option) should also be further investigated and might lead to considerations to possibly differentiate more clearly in item wording or combine items. The skills network approach also showed strong network connections between those three items (and items 3 and 4)^43^. Furthermore, the present study highlighted that some aspects patients assess in the SDM-Q-9 might not actually be known to them (e.g., if they received complete information about pros and cons, item 4 (informing on options)).

Regarding content anchors for highest and lowest ratings, variation between participants was substantial. This might imply that the same actual situation and physician behavior might receive a high score from some, but a low score from other raters. If so, direct conclusions from patient-reported SDM-Q-9 scores to actual physician SDM competence need to be handled with care. Quantitative results support this finding by showing low correlations and therewith a lack of convergent validity between the SDM-Q-9 and the Observer OPTION12 tool^16^. The skills network approach might again be a solution to this and could predict observer-rated SDM competence from the pattern of association of SDM skills^43^.

We found variability between the underlying theoretical construct, the SDM-Q-9 items, and participants’ interpretations for items 2 (sharing the decision), 5 (supporting comprehension), and 6 (eliciting preferences). This was explained partially by differences between the underlying theory and the item wordings and partially by how participants interpreted the items. Furthermore, as we found several prerequisites and difficulties with the SDM-Q-9, we suggest to interpret SDM-Q-9 scores with as much context information as possible (e.g., by using the information for the introductory questions, other clinical information, or triangulating assessment perspectives). If a consultation was for example a routine check-in appointment, answering the SDM-Q-9 seems not advisable.

It is important to note that this study should be the first step followed by similar studies in other contexts, before evidence-based recommendations about how and when to use SDM-Q-9 is appropriate. Nevertheless, these first results suggest that revising SDM-Q-9 should be considered and clear guidance on if, when, with which populations, and how to use it should be developed, as discussed above.

The structured approach assessing each item separately and in depth is a major strength of this study. Also, we chose to directly ask patients with cancer (i.e., a subsample of the target group), as recommend by, for instance, the COSMIN criteria^26^. Since this is a patient-reported measure, we thought it indispensable to understand how patients interpret each item and whether their interpretations match the underlying theoretical construct. However, several limitations have to be considered. This study’s methodology does not allow conclusions about the significance and relevance of its results across the entire population of patients with cancer or for people with other health problems. The results of this study are first indications and interpretations need to be handled with caution. Generalizability is limited given the relatively small sample of patients with cancer from one comprehensive cancer center. Men and participants with low health literacy were over-represented in the study sample. Statistical associations between gender and/or health literacy and the response process are possible. Future studies should include larger samples of patients with more diverse demographic and clinical characteristics and assess such possible associations. As health literacy and shared decision-making (as measured by the SDM-Q-9) are both rather cognitive-rational constructs, one could investigate if there is a minimal level of health literacy needed to be able to answer the SDM-Q-9 with validity. Overall, the hypotheses generated from our data need further investigation, before revision of the measure should be considered.

In conclusion, this qualitative study assessing the response process validity of the SDM-Q-9 from the perspective of patients with cancer showed good results for most items. However, it also generated hypotheses about difficulties (especially regarding necessary prerequisites) and misinterpretations (especially regarding item 2) that warrant further investigation. This study showed that even for well-established and frequently used measures such as the SDM-Q-9, the assessment of response process validity is crucial for effective assessment of SDM.

## Electronic supplementary material

Below is the link to the electronic supplementary material.

Supplementary Material 1

## Data Availability

Deidentified data that support the findings of this study are available on reasonable request. Investigators who propose to use the data have to provide a methodologically sound proposal directed to the corresponding author. Signing a data use/sharing agreement will be necessary, and data security regulations both in Germany and in the country of the investigator who proposes to use the data must be complied with. Preparing the data set for use by other investigators requires substantial work and is thus linked to available or provided resources.
