# Supplementary material for: Response process validity of the 9-item shared Decision-Making Questionnaire (SDM-Q-9) in a cognitive interview study with patients with cancer
Source: Sci Rep. 2025 May 3;15:15479. doi: 10.1038/s41598-025-99640-2 (PMC12049467; doi:10.1038/s41598-025-99640-2)
Supplement: Supplementary file 1 — Supplementary Material 1 [file 41598_2025_99640_MOESM1_ESM.pdf]

Response process validity of the 9-item Shared Decision-Making Questionnaire (SDM-Q-9) – A cognitive interview study with patients with cancer

Hannah Sigl[1,], Levente Kriston [1,], Isabelle Scholl [1,], Martin Härter [1,], Pola Hahlweg [1]

[1] Department of Medical Psychology, University Medical Center Hamburg-Eppendorf, Hamburg, Germany

Corresponding author: Dr. Pola Hahlweg (E-mail: p.hahlweg@uke.de)

Supplementary Material

- Additional file 1: COREQ checklist
- Additional file 2: COSMIN reporting guideline
- Additional file 3: Interview guide
- Additional file 4: Coding scheme and exemplary quotes

COREQ (COnsolidated criteria for REporting Qualitative research) Checklist

Note: The page numbers in this document refer to the initially submitted version of the manuscript.

Topic	Item No	Guide Questions/Description	Reported on page No.
**Domain 1: Research team and reflexivity**			
Personal characteristics			
Interviewer/ facilitator	1	Which author/s conducted the interview or focus group?	7
Credentials	2	What were the researcher's credentials? E.g. PhD, MD	7
Occupation	3	What was their occupation at the time of the study?	7
Gender	4	Was the researcher male or female?	7
Experience and training	5	What experience or training did the researcher have?	7
Relationship with participants			
Relationship with participants	6	Was a relationship established prior to study commencement?	7
Participant knowledge of the interviewer	7	What did the participants know about the researcher? e.g. personal goals, reasons for doing the research	7
Interviewer characteristics	8	What characteristics were reported about the interviewer/facilitator? e.g. Bias, assumptions, reasons and interests in the research topic	7
**Domain 2: Study design**			
Theoretical framework			
Methodological orientation and Theory	9	What methodological orientation was stated to underpin the study? e.g. grounded theory, discourse analysis, ethnography, phenomenology, content analysis	8
Participant selection			
Sampling	10	How were participants selected? e.g. purposive, convenience, consecutive, snowball	8

Method of approach	11	How were participants approached? e.g. face-to-face, telephone, mail, email	8
Sample size	12	How many participants were in the study?	9
Non-participation	13	How many people refused to participate or dropped out? Reasons?	9
Setting			
Setting of data collection	14	Where was the data collected? e.g. home, clinic, workplace	8
Presence of non-participants	15	Was anyone else present besides the participants and researchers?	8
Description of sample	16	What are the important characteristics of the sample? e.g. demographic data, date	9-10 and Table 2
Data collection			
Interview guide	17	Were questions, prompts, guides provided by the authors? Was it pilot tested?	8 and Additional file 3
Repeat interviews	18	Were repeat interviews carried out? If yes, how many?	8
Audio / visual recording	19	Did the research use audio or visual recording to collect the data?	8
Field notes	20	Were field notes made during and/or after the interview or focus group?	8
Duration	21	What was the duration of the interviews or focus group?	9-10
Data saturation	22	Was data saturation discussed?	15
Transcripts returned	23	Were transcripts returned to participants for comment and/or correction?	8
**Domain 3: analysis and findings**			
Data analysis			
Number of data coders	24	How many data coders coded the data?	9
Description of the coding tree	25	Did authors provide a description of the coding tree?	Additional file 4

Derivation of themes	26	Were themes identified in advance or derived from the data?	8-9
Software	27	What software, if applicable, was used to manage the data?	9
Participant checking	28	Did participants provide feedback on the findings?	9
Reporting			
Quotations presented	29	Were participant quotations presented to illustrate the themes/findings? Was each quotation identified? e.g. participant number	no
Data and findings consistent	30	Was there consistency between the data presented and the findings?	9-14 and Additional file 4
Clarity of major themes	31	Were major themes clearly presented in the findings?	9-14 and Additional file 4
Clarity of minor themes	32	Is there a description of diverse cases or discussion of minor themes?	9-14 and Additional file 4

Tong A, Sainsbury P, Craig J. Consolidated criteria for reporting qualitative research (COREQ): a 32-item checklist for interviews and focus groups. International Journal for Quality in Health Care. 2007. Volume 19, Number 6: pp. 349 – 357

COSMIN reporting guideline

From Gagnier JJ, Lai J, Mokkink LB, Terwee CB. COSMIN reporting guideline for studies on measurement properties of patient-reported outcome measures. *Qual Life Res*. 2021;30(8):2197-2218. doi:10.1007/S11136-021-02822-4

**General reporting recommendations relevant for all studies on measurement properties**			
Item no.	***Item name***	***Item description***	***Page***
Report section: title			
T1	Patient-reported outcome measure (PROM)	The name of the PROM instrument(s) (and version if relevant) being studied	1
T2	Measurement property (MP)	What MPs are being studied or more generally, that MPs are being studied (if there are many properties being investigated, for example)	1
T3	Study sample	General description of relevant study sample characteristics (e.g., condition of interest, language) and also any intervention or exposure (e.g., treatments) if applicable	1
Report section: abstract			
A1	PROM	The name of the PROM instrument(s) (and version if relevant) being studied (i.e., the SF-36 or SF-12; language version) or if it concerns an item bank (e.g., PROMIS instruments). The type of instrument (e.g., a self-reported questionnaire or interview)	2
A2	Measurement property	What MPs are being studied or more generally, that MPs are being studied (if there are many properties being investigated, for example)	2
A3	Design	The type of study being used to test the properties (e.g., test–retest design, longitudinal study, cohort, cross sectional, case series, randomized etc.). Other details of the study design if relevant (intervention/exposure, description of comparison instruments, outcomes other than PROMs)	2
A4	Sample	Inclusion / exclusion criteria. General description of relevant study sample characteristics (e.g., condition of interest, geographic location, language, other relevant demographic and baseline characteristics)	2
A5	Methods	A brief description of the methods for investigating each MP including statistical analyses	2
A6	Results	The main results for all MPs investigated reporting statistics for each result with measures of precision where appropriate	2
A7	Discussion/ Conclusions	A brief description of the results in the context of existing evidence, main strengths and drawbacks and the need for future research on the PROM(s) investigated	2
Report section: introduction			
I1	Name and describe the PROM of interest	Specify the name, type, language, and version of the PROM being investigated and how it was developed. Describe the construct the PROM aims to measure and its subscales; describe the structure of the PROM (e.g., the number of factors, the number of items, scoring algorithm); describe relevant instructions (like time period), and number or type of response categories. State whether the PROM is based on a reflective or formative model<br>Note: This information may also appear in the methods section in greater detail	4
I2	Target population	Describe the specific target population that the PROM was designed for. The authors need to provide the appropriate and necessary characteristics of this population	n/a
I3	Citation for the original development of the PROM	The citation for the original development paper(s) should be provided and other highly relevant citations related to the quality of the specific PROM under investigation	4
I4	State of knowledge & Rationale	A description of the current scientific knowledge (what is known) regarding the MPs of the PROM under investigation. The authors should	4

		provide a literature review or refer to a recent review of all existing evidence of the specific version (e.g., language, short form) of the PROM and explain why the new study is necessary and important. The rational for the current proposed study should be given	
I5	Definitions	Specialized terms should be defined or explained	4
I6	Objectives and hypotheses	State the specific objective(s) of the research and hypotheses related to the specific PROM under investigation	5
Report section: general methods			
GM1	Study design	State the key elements of the study design	6
GM2	Participants	State how the participants were chosen; the inclusion and exclusion criteria. (e.g., if a PROM for a specific condition, then the eligibility and selection criteria should reflect this)	6
GM3	PROM administration	An explicit description of how and when the PROM(s) were administered (e.g., in what setting) including data collection devices/system used (e.g., paper-based, electronic administration / ePRO) should be provided	7
GM4	Data collection procedures	Provide information about other data collection, exposure methods (e.g., allocation to interventions) and time points / follow-up points	6-7
GM5	Power/sample size calculation	Provide a power calculation for all MP analyses. Alternatively, if a rule of thumb is used, state it and the source/citation	6-7
GM6	Statistical analyses	Statistical analyses and tests corresponding to all hypotheses or objectives for all MPs should be reported. Where appropriate, a cutoff for statistical significance should be reported (e.g., p-value less than 0.05). A description of all statistics to be used to estimate the magnitude and direction of effect should also be reported, together with measures of variability or precision. Report statistical package used.	7-8
GM7	Missing data	State approaches or plan for dealing with missing data	n/a
GM8	Post hoc analysis	The report should specify analyses that used data after the data collection period concluded (i.e., if the analyses were post hoc; secondary data analyses) and describe the rationale for any post hoc analyses	n/a
Report section: general results			
GR1	Missing data	The amount and reasons for missing data should be explained for all analyses for all PROMs (or other outcome measurement instruments) and relevant groups	n/a
GR2	Participant/patient Characteristics	The study patients' characteristics should be described, including baseline PROM scores	8
GR3	Sample size	If one study contained analyses using different sample sizes, the authors should report the sample size for each analysis	n/a
Report section: discussion			
D1	MP evidence	Per measurement property the authors should compare the result to the criteria for good measurement properties (e.g., COSMIN criteria) [25], and determine if the specific MP is sufficient or not. Note: This information may also appear in the results section in greater detail in a table for example	??
D2	Practical relevance	The authors need to discuss the practical relevance of the findings	12-15
D3	Strengths and limitations	Strengths and limitations of the study should be discussed. For example, discuss if there were any significant potential biases in the study that could have impacted the results	12-13
D4	Generalizability	Generalizability issues related to the PROM results should be discussed. For example, discuss if the results could be generalized to other populations given the sample studied	13
D5	Instrument changes	Discuss the need for modifications to the existing PROM or new PROM development. If you conclude that one of the measurement properties is insufficient, you could suggest some modification, or if it is really poor, you could suggest stopping use of the PROM (in the specific population or in general)	12-15

D6	Future research	Report specifically the type of research needed to answer new questions arising out of these findings for the particular MP and PROM investigated	12-15
Report section: conclusions			
C1	Conclusions	State the overall conclusions for each MP and of the use PROM investigated	15
Report section: other information			
O1	Conflict of interest	State any relevant conflict of interest related to the PROM under investigation (e.g., an author being the PROM developer, funding body etc.)	16
Specific reporting recommendations for studies on content validity			
CV1	Relevance	Report if and how patients and/or professionals were asked whether each item is relevant for their experience with the condition	n/a
CV2	Comprehensiveness	Report if and how patients and/or professionals were asked whether all key concepts are included	n/a
CV3	Comprehensibility	Report if and how the comprehensibility of the PROM instructions, items, response options, and recall period was assessed	n/a
CV4	Relevance results	Report if all items were considered relevant for the construct, population, and context of use of interest by patients and/or professionals	n/a
CV5	Response options and recall period	Report whether the response options and recall period were considered appropriate by patients and/or professionals	n/a
CV6	Comprehensiveness results	Report whether patients and/or professionals considered all key concepts to be included in the PROM	n/a
CV7	Comprehensibility results	Report whether patients understood the PROM instructions, items, and response options as intended	n/a
Specific reporting recommendations for studies on structural validity			
SV1	Factor analyses: classical test theory (CTT) PROMs	Report details of the methods and results for any exploratory or confirmatory factor analyses. State the rational for any explorative factor analyses (e.g., no clear a priori hypotheses). For CFA, describe and justify the factor structure of tested models. Methods and results for checking of the assumptions should be described, the method of estimation, goodness-of-fit statistics and cutoff points for good model fit, including factor loadings of best-fitting model	n/a
SV2	Item Response theory (IRT) analyses	Type of IRT/Rasch model should be reported. Also report the method of estimation, methods and results for checking of the assumptions (unidimensionality (see factor analysis), local dependency (e.g., residual correlations), monotonicity; (e.g., Mokken scaling), goodness-of-fit statistics, and cutoff points for goodness of item/model fit, and all item parameters	n/a
Specific reporting recommendations for studies on internal consistency			
IC1	Unit of measurement	Report internal consistency methods and results for each unidimensional scale or subscale. Report all evidence or assumptions associated with unidimensionality	n/a
IC2	Continuous scores	Report Cronbach's alpha or omega statistics. Report other statistics calculated for internal consistency of continuous scores	n/a
IC3	Dichotomous scores	Report Cronbach's alpha or Kuder–Richardson coefficient. Report other statistics calculated for internal consistency of dichotomous scores	n/a
Specific reporting recommendations for studies on cross-cultural validity\measurement invariance			
CCV 1	Comparator group(s)	Report characteristics of (sub)groups being compared. Include sample sizes in each group	n/a
CCV 2	Factor analyses: classical test theory (CTT) PROMs	Report details of the methods and results for multiple-group confirmatory factor analyses, logistic regression analyses, or other analyses performed. Describe and justify the series of tested models, including constraints of factor loadings, intercepts and variances in CFA. Methods and results for checking of the assumptions should be described. Criteria to define invariance. Describe the method of estimation, goodness-of-fit statistics and criteria used to flag items for measurement invariance	n/a

CCV 3	Item response theory (IRT) analyses	Type of IRT/Rasch model should be reported. Also report the methods and results for checking of the assumptions (unidimensionality (see factor analysis), local dependency (e.g., residual correlations), monotonicity; (e.g., Mokken scaling). Describe statistical packages, method of estimation, criteria used to flag items for DIF, and methods and results of all model comparisons	n/a
Specific reporting recommendations for studies on reliability			
R1	PROM administrations	Report the total number of measurements made and if the measurements were applied to the same samples using the same PROM. The process of administrating the measurements to the patients should be described, including who administered it (i.e., did the patient complete it or was there a proxy), when, how and any time intervals between administrations should be reported. This should include: time interval between repeated measurements (e.g., was the patient stable or not), the test type (e.g., a self-administered questionnaire, an interview-based PROM), the setting in which the instrument was administered (e.g., at the hospital, or at home), and the instructions given for completing it. If relevant, other instruments or measurements accompanying the repeated PROM measurement. Also, if relevant, the independence (whether the PROM was completed without knowledge of the previous scores) of the administrations	n/a
R2	Statistical analyses	All statistical analyses and results specific to the reliability assessment(s) should be described and their use justified (e.g., the intraclass correlation coefficient (ICC) model or type of Kappa coefficient used). Also, describe the variance components, and the weighting scheme used for ordinal scores (e.g., linear or quadratic weights)	n/a
R3	Methods to improve reliability	Report any methods used to improve reliability such as restriction of the sample, training of researchers and standardization of methods, and averaging of repeated measurements	n/a
Specific reporting recommendations for studies on measurement error			
ME1	PROM administrations	Report the total number of measurements made and if the measurements were applied to the same samples using the same PROM. The process of administrating the measurements to the patients should be described, including who administered it (i.e., did the patient complete it or was there a proxy), when, how and any time intervals between administrations should be reported. This should include: time interval between repeated measurements (e.g., was the patient stable or not), the test type (e.g., a self-administered questionnaire, an interview-based PROM), the setting in which the instrument was administered (e.g., at the hospital, or at home), and the instructions given for completing it. If relevant, other instruments or measurements accompanying the repeated PROM measurement. Also, if relevant, the independence (whether the PROM was completed without knowledge of the previous completion) of the administrations	n/a
ME2	Statistical analyses	All statistical analyses and results specific to measurement error assessment(s) should be described and their use justified. Specifically, for continuous scores report the Standard Error of Measurement (SEM; Specify the exact model used to calculate the SEM (i.e., SEM consistency or SEM agreement)), Smallest Detectable Change (SDC; specify formula used, included the model of the SEM when based on the SEM) or Limits of Agreement (LoA). For dichotomous/nominal/ordinal scores report marginals (raw data) and the percentage specific (e.g., positive and negative) agreement	n/a
Specific reporting recommendations for studies on criterion validity			
CriV 1	Criterion	Report the details of the criterion used and why it was used. Justification of the gold standard must be reported. Also, describe (if applicable) how and why the criterion was dichotomized or classified. Also, how and when the criterion was administered (e.g., if independent from the PROM)	n/a

CriV 2	Continuous scores	Report correlations (when criterion has continuous scores) or the area under the receiver operating characteristic (ROC) curve (when criterion is dichotomous)	n/a
CriV 3	Categorical scores	Described how (and why) the PROM was dichotomized or made into multiple categories. Report sensitivity and specificity statistics	n/a
Specific reporting recommendations for studies on hypotheses testing for construct validity			
ConV 1	Comparator instrument(s)	The comparator instruments should be appropriately described in terms of the construct(s) they intend to measure. Report the measurement properties of the comparator instruments and related citations or data	n/a
ConV 2	Comparator Group(s)	Report characteristics of groups being compared. Include sample sizes in each group	n/a
ConV 3	Hypotheses	Report all hypotheses including the direction and magnitude of the expected correlations between the PROM of interest and another measurement instrument, or the direction and magnitude of differences in scores of the PROM between groups	n/a
ConV 4	Statistical analyses	Report all statistical methods and results used to test each hypothesis	n/a
ConV 5	Results	Report which specific results are in accordance with its hypothesis	n/a
Specific reporting recommendations for studies on responsiveness			
Resp 1	Comparison instrument(s)	The comparator instruments should be appropriately described in terms of the construct(s) they intend to measure. Report the measurement properties of the comparator instruments and related citations or data	n/a
Resp 2	Comparator group(s)	Report characteristics of groups being compared. Include sample sizes in each group	n/a
Resp 3	Hypotheses	Report all hypotheses including the direction and magnitude of the expected correlations between changes in the PROM of interest and change in another measurement instrument, or the direction and magnitude of differences in change scores of the PROM between groups	n/a
Resp 4	Measurement procedures	Report if measurements were applied to the same sample using the same instruments. Describe the measurement procedures, including time intervals between different measurement instruments	n/a
Resp 5	Interim period	The interim period between time points should be described	n/a
Resp 6	Intervention/ exposure	Describe the intervention given or exposure in the interim period if relevant	n/a
Resp 7	Patients changed	Report the proportion of patients that improved or deteriorated (and the details of any anchor used) on the construct measured on all PROMs. Report any changes in scores of the PROM in the target population for the research application relative to the predefined hypotheses	n/a
Resp 8	Statistical analyses	Report all statistical methods and results used to test each hypothesis	n/a
Resp 9	Results	Report which specific results are in accordance with its hypothesis	n/a

INTERVIEW GUIDE FOR COGNITIVE INTERVIEWS TO ASSESS THE RESPONSE PROCESS VALIDITY OF THE SDM-Q-9

PREPARATION OF THE INTERVIEWS / EQUIPMENT

- 2 audio recording devices
- Interview guideline
- Participant consent form (2x, if not yet signed)
- SDM-Q-9 questionnaire
- Short survey with demographic questions, CPS, HLS-EU-Q16
- Compensation for expenses form
- Pen
- Beverages

INTERVIEW

INTRODUCTION

- Thank for participation. Offer something to drink.
- Participation consent form: If not yet signed and returned, clarify any remaining questions and collect form. If someone has not yet read the consent form, hand it out again and give time to read and sign. IMPORTANT: No interview without signed participation consent form!
- Inform about confidentiality and reporting in anonymous form only: "Everything you say during the interview will be reported in anonymous form only. If names or other personal information are mentioned during the interview, these will be anonymized during the transcription process."
- Seek additional verbal agreement to audio recording before turning recording device on.
  → TURN ON RECORDING DEVICE!
- Information about the interview:
  - Duration: approximately 60 minutes
  - Inform about short anonymous demographic survey at the end.
  - Option to cancel: "You can cancel the interview at any time without incurring any disadvantages."
- Brief introduction: "Here at the Department of Medical Psychology, one of our research focuses is how to improve the organization of health care. As you know, my name is XX and I conduct the interview today as part of my master thesis."
- Topic SDM-Q-9: "With the questionnaire at hand, we are taking a closer look at patient-physician-encounters, especially regarding treatment decisions. Patients fill out the questionnaire after the encounter

and assess to what extent decisions were shared. This can help us to improve patient-physician-encounters, if necessary."

- Aim of the interview: "To better understand how you arrive at your answer; to understand why you tick a certain response option."
- No right or wrong: "There is no right or wrong during the interview – neither regarding the process nor the final response. We are interested in how you arrive at your answer. Every detail of your thoughts can therefore be very helpful for us. Feel free to say everything you think. Your personal opinion is important to us."

EXPLANATION OF THE INTERVIEW PROCESS

- The interview will have two parts.
- First part: Fill in the questionnaire while thinking aloud. "Let your thoughts run free, so I can try to follow your understanding of the questions and how you arrive at your response. Once again: There is no right, no wrong, every detail is important."
- Second part: Probing "I will ask specific questions to understand even better how you proceeded."

FIRST PART

"I will now hand out the questionnaire to you and ask you, as I said, to fill it out. Take as much time as you need and please think aloud."

SECOND PART

SDM-Q-9 Items:

1. *My doctor made clear that a decision needs to be made.*
2. *My doctor wanted to know exactly how I want to be involved in making the decision.*
3. *My doctor told me that there are different options for treating my medical condition.*
4. *My doctor precisely explained the advantages and disadvantages of the treatment options.*
5. *My doctor helped me understand all the information.*
6. *My doctor asked me which treatment option I prefer.*
7. *My doctor and I thoroughly weighed the different treatment options.*
8. *My doctor and I selected a treatment option together.*
9. *My doctor and I reached an agreement on how to proceed.*

Response options: *completely disagree / strongly disagree / somewhat disagree / somewhat agree / strongly agree / completely agree*

For each item the following probes were asked:

- Please restate this sentence in your own words.

- OPTIONAL (if not yet explained in sufficient detail): How did you arrive at your response?
- Please describe a situation where you would have chosen the response option "completely agree".
- Please describe a situation where you would have chosen the response option "completely disagree"
- Was this item easy or difficult for you to answer?

General question at the end:

"Now we have looked very closely at the individual questions. If you now look at the entire questionnaire again: In your opinion, which of the individual questions or behaviors are particularly important for joint decision-making? Please feel free to answer this spontaneously."

CLOSING

Final question: "Is there anything else you would like to add? Something that is important to you?"

- Hand out short survey on demographic data
- Collect questionnaires and compensation for expenses form
- Thank for participation and send off participant

Do not turn off recording device before participant left the room!

Sigl et al. Response process validity of the 9-item Shared Decision-Making Questionnaire (SDM-Q-9) – A cognitive interview study with cancer patients.

Additional file 4. Coding scheme.

Legend: Int=Interviewee; HS=Hannah Sigl (Interviewer); P=prerequisite; I=interpretation; D=difficulty.

ID	Codelevel 1	Codelevel 2	Codelevel 3	Memo	n(codings)	n(documents)	Quotes
	**Total**				452	11	
00	**General comments**						
00.1		Difficulties rating one specific clinical encounter only		Participants reported difficulties to answer the questionaire with only one specific encounter in mind.	7	6	**Interview 04 on the introductory questions:** Interviewee (Int): "The consultation to make a decision actually took place on two occasions. A different treatment was originally planned, but I wasn't suitable for it due to certain parameters, so to speak. That's why we jointly decided on an alternative therapy."
00.2		Prerequisite: Having options and a choice		Participants voice that they can only answer the questionnaire if there is more than one treatment option to choose from.	4	4	**Interview 06, at the end:** Int: "I believe that I was able to answer a few questions about my case in a relatively trivial way, because I don't have any options in terms of different treatment options, different procedures or preference for treatment options."
00.3		Jargon "Partizipative Entscheidungsfindung"		Participants had questions what "Partizipative Entscheidungsifndung" (SDM in German) meant and thought is was difficult to understand.	3	3	**Interview 04, in the beginning:** Int: "The established term "partizipative Entscheidungsfindung" exists in medicine and is seen as shared decision-making between patient and doctor, but I don't think everyone grasps the term at first."
00.4		Need for explanation		Participants thought rating on questionnaire to be limited in its meaningfulness and wanted to explain themselves.	2	2	**Interview 06, while filling in questionnaire:** Int: "To be honest, that's actually bad. So from that point of view: I'm going to write "rather not true" here." - I: "Mm-hm." - P: "With what I said as an explanation."
00.5		Questionnaire not cancer-specific		One participant criticized the questions in the questionnaire being to vague and would have preferred more specific questions for the context of cancer care.	1	1	**Interview 08, at the end:** Int: "About the questionnaire: My question would be, the others who come here, do they all have relatively the same clinical situation?" - HS: "Well, I only speak to oncology patients at the moment." - Int: "Then maybe that the questions could be a bit more specific."
01	**Item prerequisites, interpretations, and difficulties**			Concrete statements that describe how the participant understands each item.			
01.1		Interpretation item 1 (focusing the decision)		Item 1: *My doctor made clear that a decision needs to be made.*			
01.1. P1			Prerequisite (P): Having options and a choice	Participants voiced that they can only answer item 1 if there is more than one treatment option to choose from.	4	4	**Interview 03**: Int: ""I have cancer and in my case there is no real consideration or variation on how to do it all. Because in this case you definitely have to do therapy and - in my case - the physicians had a direction of therapy and said that we would do so and so and so.""
01.1.I1			Interpretation (I): Decision-making necessity	Participants perceived an emphasis on the need to make a decision. In the German SDM-Q-9 the wording is that a decision "has to be" made. This need to make a decision has to be explicitly voiced by the physician.	9	9	**Interview 02:** Int: "That something had to happen in any case, because if it continued like this, it would have gone totally negative. Then we wouldn't have been able to help you any further, that's about it. If you agreed, you had a chance. That's how I actually see it."
01.2		Interpretation item 2 (sharing the decision)		Item 2: *My doctor wanted to know exactly how I want to be involved in making the decision.*			
01.2.P1			P: Having options and a choice	Participants voiced that they can only answer item 2 if there is more than one treatment option to choose from.	8	7	**Interview 06:** Int: "I couldn't take part in the decision because there was no alternative. I wouldn't have had the opportunity to change anything. So not even 5% or anything like that. I had to do 100% of what I was told to do."
01.2.I1			I: Possibility to be involved in decision-making	One partiicpant interpreted this item as patients having the opportunity to be involved in making the decision.	1	1	**Interview 08:** Int: "For me, it meant that I could make my own decisions or get involved in the decision-making process."
01.2.I2			I: Dialogue between patient and physician is sufficient	One participant thought it sufficient if the patient and the HCP had a dialogue about the decision. This participant did not deem it neccessary to explicitly talk about the patients desired level of participation.	1	1	**Interview 04:** Int: "This question did not even arise as to whether I wanted to be involved, but I was directly involved through the dialog."
01.2.I3			I: Physician asked for patient opinion	One participant interpreted that the HCP explains the situation and asks the patient what their opinion is and if they agree.	1	1	**Interview 05:** Int: "My physician wanted me to explain her findings to me, so to speak, and then asked me what I thought of them and what options I saw and whether I thought the same as she did."
01.2.I4			I: "How" to make the decision	One participant voiced that this item asked for the "how" of making a decision.	1	1	**Interview 03:** Int: "Yes, that's how I decide on a certain type of treatment, isn't it?"
01.2.D1			Difficulty (D): Did not occur / does not apply	Participants reported that physicians asking about this did not occur and therewith the question does not apply.	5	4	**Interview 06:** Int: "So nobody asked me if I wanted to be involved, but it was basically clear to everyone that it had to be done. I mean, what should I do?"
01.2.D2			D: Prerequisite not met	If the prerequisite was not met, participants had difficulties answering this item.	2	2	**Interview 06:** Int: "If I have a cut on my head, I can always say: "No, I'll put a band-aid on it and go home." But not in my situation, of course. So when I say "completely true", that's not really true because I wasn't asked about it."

01.2.D3	D: Limited understanding of types of involvement	Participants did not comprehend properly what different possibilities of involvement in decision-making could be. They also did not perceive it as having a choice in how they wanted to be involved, but rather as pre-decided or not needing a decision.	2	2	**Interview 07:** Int: "How should I participate now? How should I decide? For example, what is my involvement in this? I don't understand that."
01.2.D4	D: Need for "no options/choice" (="does not apply")	One participant voiced that having the option to check "no options/choice" would be helpful.	1	1	**Interview 06:** Int: "The answer option "no alternative" would also be conceivable, so to speak." HS: "I understand that well, because of course you could interpret it differently without knowing..." Int: "That's exactly how it is. It has nothing to do with incapacity or organization or anything here, but unfortunately that's simply the case or predetermined due to the illness."
01.3	Interpretation item 3 (presenting options)	Item 3: *My doctor told me that there are different options for treating my medical condition.*			
01.3.P1	P: Having options and a choice	Participants voiced that they can only answer item 3 if there is more than one treatment option to choose from.	5	4	**Interview 01**: Int: "No, in that case there was nothing else. There was nothing else. Or to put it briefly: I wouldn't be sitting here anymore."
01.3.I1	I: Physician named more than one treatment option	Participants interpreted that the physician named and explained more than one treatment option.	7	7	**Interview 08:** Int: "That he gave me several options on how I can continue to treat my illness."
01.3.I2	I: Explanation of pros and cons	Participants interpreted that the physician explained pros and cons of the treatment options.	3	3	**Interview 2:** Int: "He said that fever can occur, this can occur, that can occur, different symptoms can occur. With this option, which I chose, these could have been too. But I preferred the last straw to nothing, didn't I?"
01.3.D1	D: Difficulties choosing an score	Two particpants struggeled choosing the "right" score for item 3.	4	2	**Interview 02:** Int: "Can you give an example of when this should mean "does not apply at all"? I don't know at all how to..."
01.4	Interpretation item 4 (informing on options)	Item 4: *My doctor precisely explained the advantages and disadvantages of the treatment options.*			
01.4.P1	P: Patient was receptive to information	Participants voiced that for item 4 to occur patints need to have been receptive to information. This was not always the case after having heard about a cancer disgnosis.	2	2	**Interview 06:** Int: "Because as a person affected in such a critical situation, you don't take anything in at first. So when you get a diagnosis like that, you sit there with your mouth shut and then you don't really have any more thoughts." - HS: "So it becomes a bit blurred what was actually said?" - Int: "Exactly. And of course it's not the doctor's fault. But that's logical. Anyone would feel that way."
01.4.I1	I: Explanation of pros and cons	Participants interpreted that the physician explained pros and cons of the treatment options. Information material can be used for additional explanation.	11	9	**Interview 01:** Int: "When the doctor explains the different procedures, it always has to do with the positive and negative aspects of the treatment, i.e. the advantages and disadvantages. I think that's part of the information. So I can also say it another way: What positive development is hoped for and what dangers are possibly hidden or possible in the background? That's the question behind the question."
01.4.D1	D: Incomplete information given by physician	Participants reported that sometimes physicians do not diclose all possible side effects and that e.g. information materials might list additional side effects that were not mentioned by the physician. Partiicpants also mentione dthat this might be beneficial to the patient in a difficult situation	4	3	**Interview 06:** Int: "But in retrospect, there are a lot of effects and side effects of such severe treatments that are not funny. I think you could say a bit more about that in advance."
01.5	Interpretation item 5 (supporting comprehension)	Item 5: *My doctor helped me understand all the information.*			
01.5.P1	P: Patient was receptive to information	Participants voiced that for item 5 to occur patints need to have been receptive to information. This was not always the case after having heard about a cancer disgnosis.	2	2	**Interview 06:** Int: "It [the information] no longer gets through. It's certainly different with other diseases, but when you do the survey in oncology, you're only dealing with cancer patients and everyone is in shock. There's no other way. "
01.5.I1	I: Comprehensibility of information	Participants interpreted that physicians should give information in an understadable manner to patients. This includes simple explanations and no medical jargon or latin.	7	6	**Interview 05**: Int: "Doctors sometimes have incomprehensible, Latin-influenced expressions and that's natural for them. They talk like that and it's okay that they have such a code language, but for the patients it's sometimes difficult and can also be frightening."
01.5.I2	I: Answering questions	Participants voiced that questions asked by patients need to be answered by physicians.	4	4	**Interview 09:** Int: "In other words, he answered all the questions I had about the medication, the illness and the treatment sufficiently for me at the time."
01.5.I3	I: Complete information	Participants voiced that the information given also needs to be complete.	3	3	**Interview 03:** Int: "I have complete information, in all different forms it is explained to me."
01.5.I4	I: Physician checked understanding	One participant voiced that physicians need to check patients' understanding of the given information.	1	1	**Interview 01:** Int: "I asked again whether it was received as he meant it"
01.6	Interpretation item 6 (eliciting preferences)	Item 6: *My doctor asked me which treatment option I prefer.*			
01.6.P1	P: Having options and a choice	Participants voiced that they can only answer item 6 if there is more than one treatment option to choose from. Some participants voiced that they did not perceive "no treatment" as a viable option.	8	7	**Interview 11**: Int: "Yes, that was a bit difficult for me again, because there weren't that many options at the beginning."

01.6.I1	I: Physician elicits patient preference	Participants interpreted that the physician asked for their preferences regarding the options.	4	4	**Interview 05:** Int: "So ideally the doctor would also have shown me several treatment options that would probably be equally effective in my case and he would have said: "What would you like to do? Would you like injections? Would you like pills? Would you like something else - therapies in another form?" Exactly, he would have shown me that and I would have had a choice to do A, B or C. That's how I understand the question."
01.6.I2	I: Final decision made by patient	Participants emphasized that the final decision should lie with the patient.	3	3	**Interview 08:** Int: "Yes, that's how I understood it: In the end, he left it up to me to decide which medication I wanted to take."
01.6.D1	D: Similarities between item 6, 7, 8	Participants voiced difficulties differentiating between items 6, 7, and 8.	2	1	**Interview 01:** Int: "Questions 6, 7 and 8 are very similar, I would say. This requires linguistic knowledge and sensitivity and I would at least change the order. Perhaps you could make two questions out of three."
01.7	Interpretation item 7 (deliberating the decision)	Item 7: *My doctor and I thoroughly weighed the different treatment options.*			
01.7.P1	P: Having options and a choice	Participants voiced that they can only answer item 7 if there is more than one treatment option to choose from.	3	3	**Interview 11:** Int: "I can only give you an answer because I had an alternative. If I hadn't had one, I wouldn't have been able to answer your question at all."
01.7.I1	I: Weighing treatment options	Participants interpreted that different treatment options were weighed for the individual case of this patient.	8	8	**Interview 03:** Int: "Yes, that several treatment options are discussed and then one is selected and then thoroughly reviewed. Then it is this point."
01.7.I2	I: Trust between patient and physician	One participant voiced that having trust in the physician was essential, as patients often have to go by the information and recommendations given by the physician when weighing options.	1	1	**Interview 04:** Int: "And I have to say, as a patient who has no deeper insight into the chances of success of the drug, you have to be honest, you are actually dependent on the instructions, the recommendation from the doctor, because it is also difficult to extract something from forums on the Internet or to find out about it. There are also different therapeutic approaches and therefore a basis of trust with the treating doctor is definitely very, very important."
01.7.D1	D: Similarities between item 6, 7, 8	Participants voiced difficulties differentiating between items 6, 7, and 8.	5	3	**Interview 02:** Int: "Or here [questions] 7 and 6 are also very similar. Which treatment do I prefer and which treatment option - weigh up."
01.8	Interpretation item 8 (selecting an option)	Item 8: *My doctor and I selected a treatment option together.*			
01.8.P1	P: Having options and a choice	Participants voiced that they can only answer item 8 if there is more than one treatment option to choose from.	4	3	**Interview 03:** Int: "In my case, it was not at all possible to choose a treatment option together."
01.8.P2	P: Patient wants to be involved	One participant voiced that they did not have the energy or will to be involved in decision-making.	1	1	**Interview 02:** Int: "I just don't know what I would have done at the moment. Because you're ill and you don't have much will of your own. Have a go with me. Maybe it will work out. Yes, there is no willpower to decide, at least I don't."
01.8.P3	P: Trust between patient and physician	One participant voiced that without trust between patient and physician selecting an option together was not possible.	1	1	**Interview 02:** Int: "Ultimately, the doctors decided what to do. I was certainly asked, but not by a doctor I trusted, if you like. It was one of the treatment team who was introduced to me, but that was it. We hadn't yet reached the stage where you could build up a relationship of trust."
01.8.I1	I: Shared decision with emphasis on "together"	Participants emphasized the word "together" in item 8. Patient and physician jointly choose an option.	6	6	**Interview 08:** Int: "So for me it was like this: I told him my decision, in the end he also told me his a bit, because I gave him the option and then TOGETHER we decided in favour of the one medication for the time being."
01.8.D1	D: Similarities between item 6, 7, 8	Participants voiced difficulties differentiating between items 6, 7, and 8.	6	3	**Interview 01:** Int: "But even if you were to swap 6 and 7 and then the current 7th would be the 6th question and 6 would be the 7th question and then comes the current 8th question, then that is also very, very, very close together."
01.8.D2	D: Prerequisite not met	If the prerequisite was not met, participants had difficulties answering this item.	1	1	**Interview 06:** Int: "So we didn't choose it together, but it was clearly given to me as alternative one and the other is alternative zero. So what should I tick now? I could say "completely true" or I could also say "not true at all" depending on the situation. That's a bit difficult to be honest."
01.8.D3	D: Patient makes final decision	One participant voiced that they think the final decision has to be made by the patient and thus "together" is an inappropriate term.	1	1	**Interview 04:** Int: "Ultimately, it is the patient who makes the choice. So maybe to the question: If I look at it again, you have chosen a treatment option together. That is not right in the end, because the patient alone makes the final decision: Do I do this therapy, do I not do it?"
01.9	Interpretation item 9 (planning actions)	Item 9: *My doctor and I reached an agreement on how to proceed.*			
01.9.I1	I: Making a plan for next steps	Participants interpreted that the following steps are planned jointly by patient and physician.	11	10	**Interview 03:** Int: "Yes, that further treatment options or further steps are discussed and agreed together with the physician and patient."
01.9.I2	I: Planning alternatives in case of ineffectivene treatment	Participants also included in this item the planning of alternative treatments or ways forward in case the chosen option were not to work.	3	3	**Interview 07:** Int: "Yes, for example, if a therapy hasn't worked properly and then the physician says: "Okay, so the therapy isn't working properly. It does this and that or it has so and so many side effects and this and that. Then it would be better if we took another therapy option", so the second or third, so to speak."

01.9.D1	D: Understanding of "how to proceed"	One participant had difficulties understanding the term "how to proceed" (in German "das weitere Vorgehen").	1	1	**Interview 11:** Int: "Yes, what is an 'agreement'? The question for me was: What ia an agreement for the next steps? What kind of agreements are there? Maybe you have to think about it. I had actually said I realised that and it went well with us. But what are agreements for the next steps? It's a bit difficult, of course."
02	**Content anchors**	Concrete statements that describe situations or behaviour that would lead to them rating this item with the highest or lowest score.			
02.1	Content anchors item 1 (focusing the decision)				
02.1.H1	High: Emphasis on decision-making necessity	Physician made very clear that a decision needs to be made at this point. They emphasized the "need to be made" / "has to be made".	6	6	**Interview 05:** Int: "If, of course, he had told me openly - I don't know - if something in my illness had progressed to such an extent that I needed a - and he would have told me and said: "You have A or B as an option. We have to do something now. We have to do something now and you have to decide and I recommend that you follow my suggestion."
02.1.H2	High: Deciding against treatment was also discussed	Physcian also voiced the option to decide against treatment and choose e.g. active surveillance.	1	1	**Interview 11:** Int: "The alternative would have been to say: "Maybe you don't want to?" That wasn't actually the focus at all."
02.1.H3	High: Decision had already been made	One participant indicated the highest score as they assumed that there was decision-making necessity that led the physicians to already make a decision before the patient-physician encounter. The participant therefore deemed focusing on the decision-making necessity unneccesary.	1	1	**Interview 10:** Int: "So I wasn't even told that a decision had to be made, but was told straight away: 'We're going to do this now!' So the decision was already made in advance, because there is a diagnosis and she naturally assumes, and rightly so, that if I'm here, I want to do something and not go home and say: 'Thank you for talking to me.'" - HS: "Okay, so the decision was anticipated a bit. You had already described that, hadn't you?" - Int: "Exactly yes. Yes." - HS: "That's why you also decided in favour of "completely true", because you simply assumed..." - Int: "Exactly. Something has to happen."
02.1.L1	Low: No decision made and/or much uncertainty	No decision was made despite necessity and/or the patient remeined largely uncertain without support	3	3	**Interview 04:** Int: "If I had been dismissed, I would almost have said, with unclear delays without a decision in favour of further treatment."
02.1.L2	Low: No decision-making necessity	A decision does not need to be made.	2	2	**Interview 08:** Int: "I believe that if my values had been the same: yes, you can take some, but you don't have to take any. So as long as it's not a must to make a decision, then I would have said "doesn't apply at all", but it was actually very clear, so it's difficult to say now"
02.1.L3	Low: Physician decided without the patient	Physician made the decision without patient involvement in the decision-making process.	2	2	**Interview 07:** Int: "if the doctor had said, so to speak: "I decide. This will be this and that", without my consent, so to speak, without my agreement or insight, so to speak."
02.1.L4	Low: Prerequisite not met (no options/choice)	The prerequisite of having options and a choice was not met.	1	1	**Interview 03:** Int: "I was faced with the fact."
02.1.L5	Lowest score "impossible" (no situation or behaviour imaginable)	Participants could not imagine a situation or behavior that would have led to them indicating the lowest score.	3	3	**Interview 01:** Int: "No, I can't imagine that, because then I would have asked. Then I would have asked. So I would have asked for information and clarification. Well, I can't imagine that."
02.2	Content anchors item 2 (sharing the decision)				
02.2.H1	High: Shared dialogue	Physician involved patient in a shared patient-physician-dialogue and as an "automatic" consequence involved them in the deicison.	3	3	**Interview 04:** int: "That's why it simply emerged in the joint dialogue that a decision was necessary and we ultimately made it together."
02.2.H2	High: Physicians elicited patient preference	Physician asked patient about their opinion and preferences for themselves with their individuality in this specific situation.	3	3	**Interview 08:** Int: "If he had asked me what I wanted to do. So really me personally, what is important to me and not just: "There are such and such possibilities." Then I would have said "completely true"."
02.2.H3	Highest score "impossible" (item not understood)	Participants could not imagine a situation or behavior that would have led to them indicating the highest score as they had not undertsood the item.	2	2	**Interview 09:** Int: "No, I can't make anything out of the question. I'm sorry. "
02.2.L1	Low: Prerequisite not met (no options/choice)	The prerequisite of having options and a choice was not met.	3	3	**Interview 08:** Int: "If he hadn't given me any options. If there had only been one medication I could have taken, then it would definitely have been "does not apply at all" in that case. If I'd had no choice at all."
02.2.L2	Low: No question about decision-making preferences	Physician did not ask how the patient wanted to be involved in the decision-making process.	3	3	**Interview 05:** Int: "Yes, exactly, again - actually on all points - always reflecting on the last conversation, that we basically didn't talk about it, that she didn't ask me these questions somehow and so it wasn't relevant, so to speak."
02.2.L3	Low: Physician decided without the patient	Physician made the decision without patient involvement in the decision-making process.	3	3	**Interview 10:** Int: "Yes, you are - or I am - perhaps others feel the same way - completely incapacitated. So it's just completely decided - I'm [profession] - actually like a patient - like an animal. You go along with it."
02.2.L4	Lowest score "impossible" (item not understood)	Participants could not imagine a situation or behavior that would have led to them indicating the lowest score as they had not undertsood the item.	2	2	**Interview 07:** Int: "Exactly, but the problem is with me now: How do you involve a patient? I do not understand that."

02.2.L5		Lowest score "impossible" (no situation or behaviour imaginable, patient would prevent it)	Participant could not imagine a situation or behavior that would have led to them indicating the lowest score as they deem themselves responsible to prevent such an experience.	1	1 **Interview 01:** Int: "No, I can't imagine that either, because I would have asked or even demanded information or clarification beforehand, and without getting to know any alternatives or other options, I would not have been able to participate in the decision or would not have made a decision."
02.3	Content anchors item 3 (presenting options)				
02.3.H1		High: Physician made clear that there was more than one treatment option	The physician elaborated on more than one treatment option.	6	6 **Interview 08:** Int: "He gave me several options on how I could continue to treat my illness, let's put it that way. That's how I understood him and that's why in the end it was "completely true", because he presented me with the three options that are currently available in terms of medication and I was then able to make a decision."
02.3.L1		Low: Physician decided without the patient	Physician made the decision without patient involvement in the decision-making process.	4	4 **Interview 11:** Int: "Yes, if she had told me: "You have to take this one now and there is nothing else." Done!"
02.3.L2		Low: Prerequisite not met (no options/choice)	The prerequisite of having options and a choice was not met.	3	3 **Interview 06:** Int: "Same answer or same wording: There is no alternative for my clinical picture. Period." - HS: "Exactly, that's why you ticked 'does not apply at all'." - Int: "That's why I also ticked 'does not apply at all'. Yes, exactly."
02.3.L3		Low: No elaboration on different treatment options	Physician named different treatment options, but did not elaborate on them.	2	2 **Interview 09:** Int: "That there are options, but how they work, what side effects occur - that is what the patient is most interested in - or how long the treatment lasts. Just the information about the treatment. If that is missing, then the information has not flowed, so to speak."
02.3.L4		Lowest score "impossible" (no situation or behaviour imaginable)	Participant could not imagine a situation or behavior that would have led to them indicating the lowest score.	1	1 **Interview 02:** Int: "Can you give an example of when this should mean "does not apply at all"? I don't know at all how…"
02.4	Content anchors item 4 (informing on options)				
02.4.H1		High: Physician proactively explains pros and cons	The physician elaborated on advantages and disadvantages, benefits and risks of the options, without the patient needing to ask about them.	5	4 **Interview 08:** Int: "If he had presented the medication to me from the outset and said: "These are the advantages and these are definitely the disadvantages that can occur." If he had been able to decipher this more on his own."
02.4.H2		High: Complete information on pros and cons	Physician informed patient about all possible advantages and disadvantages, benefits and risks.	5	4 **Interview 05:** Int: "Ideally my doctor would have told me what side effects, for example, could happen if I took certain medication. And what positive effects it would probably have."
02.4.L1		Low: Superficial or no discussion of pros and cons	Physician dod not or only superficially discuss any advantages and disadvantages, benefits and risks.	7	7 **Interview 04:** Int: "If these advantages and disadvantages had only been mentioned very superficially or not at all, which would have been a bad therapy discussion for me and would not actually have provided any basis for participatory decision-making."
02.4.L2		Lowest score "impossible" (no situation or behaviour imaginable)	Participant could not imagine a situation or behavior that would have led to them indicating the lowest score.	1	1 **Interview 02:** Int: "What would have had to happen? There would have been things that I simply couldn't have imagined. I just don't know what they could have been."
02.5	Content anchors item 5 (supporting comprehension)				
02.5.H1		High: Easily comprehensible information	Physicians gave information in an easily understadable manner to patients (e.g., simple explanations, no medical jargon or latin).	4	4 **Interview 01:** Int: "The doctor has presented all the information in a reasonable and understandable manner."
02.5.H2		High: Physician answered all patient questions	All patient questions regarding the disease, diagnosis, treatment, and medication were answered by the physician.	2	2 **Interview 09:** Int: "This means that he answered all the questions I had about the medication, the illness and the treatment sufficiently for me at the time. That's why I selected "completely true"."
02.5.H3		High: Physician proactievly explains information	The physician proactively explains information given to the patient without the patient having to ask for it.	2	2 **Interview 08:** Int: "If he did that on his own more often. So maybe sometimes it's because he knows my background so well that I understand a lot and he explains a lot, definitely or informs me a lot so that I understand, but you still always have to ask a bit. If that was omitted, then it would be "completely" for me."
02.5.L1		Low: Physician does not explain information	The physician either does not give any information or does not explain the information, even after the patient asked for explanation.	2	2 **Interview 08:** Int: "If he hadn't explained anything to me, i.e. no information at all when I asked."
02.5.L2		Low: Incomprehensible information	The physician gave information that was not comprehensible to the patient.	2	2 **Interview 11:** Int: "When someone sits in front of you and explains something and doesn't even ask: "So you have to have chemotherapy now. And you have mucositis or you have - " I'm exaggerating a bit, but I'm speaking in technical jargon and then I don't understand any more of the information. So that would be "does not apply at all" to the question."
02.5.L3		Low: Insufficeint information	The physician gave insufficient information to the patient.	2	2 **Interview 03:** Int: "If I simply have the feeling that I didn't get enough information. If it wasn't really complete for me or if I say that I can't really imagine it for myself and I feel that it was wrong for me at that moment."
02.5.L4		Low: No answer to patient questions	The physician did not answer patient questions.	1	1 **Interview 09:** Int: "If my questions about the treatment, the diagnosis, the medication had actually been answered inadequately or if he had, so to speak, shot me down or whatever."

02.5.L5		Lowest score "impossible" (no situation or behaviour imaginable, patient would prevent it)	Participant could not imagine a situation or behavior that would have led to them indicating the lowest score as they deem themselves responsible to prevent such an experience.	1	1	**Interview 01:** Int: "To be honest, I can't imagine that because I wouldn't expose myself to it. I would ask. I would probe. If perhaps not at that moment, then perhaps an hour later or the next day I would ask again, saying: "There can't be just one thing in this case.""
02.6	Content anchors item 6 (eliciting preferences)					
02.6.H1		High: Prerequisite met (options/choice)	The prerequisite of having options and a choice needs to be met.	4	4	**Interview 09:** Int: "I think the wishful thinking would have been that there would have been several treatment options to choose from. There was only one, if you break it down like that, and then there were different options within the treatment option."
02.6.H2		High: Patient enabled to make decision themselves	Patients had the opportunity and felt able to make the decision themselves based on the information and possibly recommendation given by the physician.	4	4	**Interview 07:** Int: "That the physician says: "There is this and that treatment option." And then I decide what is low-risk for me, so to speak, which treatment or which treatment is more, more successful and what risks are involved, and I also decide for myself, so to speak, that I prefer this or that."
02.6.H3		High: Prerequisite not met (no options/choice)	The prerequisite of having options and a choice was not met.	1	1	**Interview 04:** Int: "Is not, I say, applicable, is not given here. In this respect, it is completely true for me, because I say I would have followed the advice, the medical advice, and in this respect I would tick "completely true" here."
02.6.H4		High: Physician asked for patient preferences	The physician explicitly asked got the patient's treatment preferences.	1	1	**Interview 05:** Int: "Ideally, the doctor would have shown me several treatment options that would probably be equally effective in my case and said: "What would you like to do? Would you like injections? Would you like pills? Would you like something else - therapies in another form?" Exactly, he would have pointed that out to me and I would have had a choice. I do A, B or C. That's how I understand the question."
02.6.L1		Low: Physician decided without the patient	Physician made the decision without patient involvement in the decision-making process.	3	3	**Interview 04:** Int: "Yes, so a course of treatment from the doctor's side without consulting me or asking whether I wanted it at all would have led to such a decision. So this question doesn't apply at all."
02.6.L2		Low: Prerequisite not met (no options/choice)	The prerequisite of having options and a choice was not met.	3	3	**Interview 06:** Int: ""Doesn't apply at all", because there is no alternative."
02.6.L3		Low: No acceptance of patient-preferred option	The physician did not accept the option the patient preferred.	1	1	**Interview 07:** Int: "If the doctor had not accepted my request for treatment. Then I would have said "does not apply at all"."
02.6.L4		Low: No information by physician	The physician did not offer information to the patient and thus no shared decision-making is possible.	1	1	**Interview 05:** Int: "If, as already mentioned in other points, the doctor had not considered this at all, so to speak. If you don't get any information, you can't make a decision. If only route 1 is ever chosen, right?"
02.6.L5		Lowest score "impossible" (no situation or behaviour imaginable if only one option)	Participant could not imagine a situation or behavior that would have led to them indicating the lowest score as they perceived having only one option.	1	1	**Interview 11:** Int: "That is difficult. I can't answer "doesn't apply at all" because there is no second option."
02.7	Content anchors item 7 (deliberating the decision)					
02.7.H1		High: Weighing of treatment options	Different treatment options are weighed for this individual case.	3	3	**Interview 07:** Int: "Yes, that is also different treatment options thoroughly weighed up to know - between these treatments that are possible and then you weigh up, so to speak, where do we go, in which direction. So do we take this and then we have such and such a high success rate, or do we take this and then the success rate is not so high, but then we have a bit of peace and quiet, or do we take this and then the risk is high, but then we have the situation where you may be free of treatment afterwards."
02.7.H2		High: Open and proactive physician communication	The physician communicates openly and proactively about the different treatment options.	2	2	**Interview 05:** Int: "If I had participated, so to speak. If the physician pointed out what treatment options were available, and maybe there was only one, right? But that she communicates this openly. Exactly, then I would have ticked "completely true"."
02.7.H3		High: Physicians weighed options at tumor conference	One partiicpant chose the highest score as they assumed that the physicians at the tumor conference must have throughly weighed the options. The patient was not involved in this process.	1	1	**Interview 10:** Int: "So it's been - well, "and I" perhaps not - but they've already weighed it up thoroughly by the fact that they sat down together in the consultation and talked about it. And if it's in the tumor board and there are different treatment options, I assume that they've thoroughly discussed with several people what would be best." - HS: "That's why you ticked "completely true"?" - Int: "Exactly."
02.7.H4		High: Equal responsibility for the decision	One participant interpreted "shared" as equal responsibility for the decision carried by the patient and the physician.	1	1	**Interview 09:** Int: ""Fully applies" would have been if we had weighed it up 50:50, so to speak."
02.7.L1		Low: No transparency in communication about options	The physician did not transparently communicate about the different treatment options.	4	4	**Interview 05:** Int: "If there had been no conversation about it. What are the possibilities somehow and what do we want to do? What do we want to do? If that doesn't happen."
02.7.L2		Low: Physician decided without the patient	Physician made the decision without patient involvement in the decision-making process.	4	4	**Interview 09**: Int: ""Does not apply at all" would have been when he [the physician] decides for me."
02.7.L3		Low: Physician decided without colleagues	Physician made the decision without involvement of colleagues in the decision-making process.	1	1	**Interview 10:** Int: "If I am presented with a treatment option that would have been decided by the physician alone. Without consulting anyone or sitting down together."
02.8	Content anchors item 8 (selecting an option)					

02.8.H1			High: Shared decision with emphasis on "together"	The emphasis is on "together". The choice for one option should be taken together based on shared information, deliberation and weighing of options.	6	6	**Interview 05:** Int: "My physician listed several treatment options for me and we discussed them openly together. What is the advantage? What are the side effects? What is best for my health? What would be the best treatment?"
02.8.H2			High: Incorporation of individual patient situation	To select an option the individual situation of this patient beyond their medical situation needs to be paid attention to and incorporated into decision-making.	1	1	**Interview 07:** Int: "The doctor gave me the therapy options and then he told me all the advantages and disadvantages of the therapy and then he also considered my living situation, where I live, where I work - my life, so to speak, my private life. And through this conversation, so to speak, therapies - there are also therapies that you can't do if you live somewhere where you have a lot of stress. Or if you're in a life situation where you're not properly adjusted, so to speak."
02.8.L1			Low: Physician decided without the patient	Physician made the decision without patient involvement in the decision-making process.	5	5	**Interview 04:** Int: "If the treatment is carried out, so to speak, on the doctor's instructions alone, without any participation or I say, yes I say, consultation or discussion with the patient, the decision is simply made."
02.8.L2			Low: Prerequisite not met (no options/choice)	The prerequisite of having options and a choice was not met. This could also apply in an emergency situation.	3	2	**Interview 03**: Int: "In my case, it was not at all possible to choose a treatment option together."
02.8.L3			Low: No incorporation of individual patient situation	The individual situation of this patient beyond their medical situation was not incorporated into the decision.	1	1	**Interview 07:** Int: "If the physician had not taken the patient's entire living situation into account, because there are many patients who live far away from here and, to be honest, they are not doing well because they don't have the opportunity to check all the parameters as regularly as we do here in [the city]."
02.8.L4			Low: Disagreement within the healthcare team	Within the healthcare team, there are different opinions about the decision.	1	1	**Interview 02:** Int: "Perhaps if I had realized that there were so many opposing opinions in this team."
02.9		Content anchors item 9 (planning actions)					
02.9.H1			High: Course of treatment elaborated	The course of treatemnt was clearly elaborated and planned.	4	4	**Interview 02:** Int: "Medication, this and that, how often I have to introduce myself here and, and. And that's why I also wrote “completely true.”"
02.9.H2			High: Planning alternatives in case of ineffectivene treatment	Alternative treatments or ways forward were planned in case of the chosen option not working.	2	2	**Interview 10:** Int: “That it was just shown how to proceed. So if it doesn't work now, what happens next or if it works and that's what was done yesterday. So if it works with the radiotherapy, what the follow-up examinations will look like and my oncologist will also tell me if it hasn't been successful that we will then tackle the next one. So the journey continues.” - HS: “Okay, that's why you also ticked ‘completely true’?” - Int: “Exactly.”
02.9.H3			High: Joint agreement on course of treatment	The course of treatemnt was jointly agreed on.	2	2	**Interview 09:** Int: "So he sort of suggested what the next steps should be and I agreed."
02.9.H4			High: Course of treatemnt tailored to individual patient situation	The course of treatment was planned in a way that it works for the patient, their wellbeing/quality of life, and their time capacities.	1	1	**Interview 08:** Int: "If I could decide when I wanted to come, roughly. Or if I notice I'm feeling worse, then I'll come and have a chat, because unfortunately it's a given that you always have a chat."
02.9.L1			Low: No information on course of treatment	The course of treatemtn remainsed unclear and uncertain.	4	4	**Interview 05:** Int: "If the physician had simply described the current situation, so to speak, and hadn't pointed out what options were available and how we wanted to proceed."
02.9.L2			Low: Rigid course of treatment	The course of treatemtn is rigid and strict. There is not room for adaptation or discussion with the patient.	2	2	**Interview 03:** Int: "That didn't apply at all in my case, or not at all, because I was simply given a fixed therapy from the start and it's still going on."
02.9.L3			Low: Late realization of ineffective treatment	The physician realized too late that a treatment did not work and did not react to this fact in a timely manner by making new plans.	1	1	**Interview 07:** Int: "For example, what if I have started therapy, right? And I'm now waiting for the physician to say: “Okay, the therapy is working. That's good, it's going well”, but if the physician is negligent, so to speak, if the doctor sees what's happened too late. Because sometimes doctors see what's happened too late and then they react too late. For me, that would also be “does not apply at all”."
02.9.L4			Low: Disagreement between patient and physician	Patient and physician disagree on the course of treatment.	1	1	**Interview 01:** Int: "When there are alternatives, but you can't come to a joint decision or something. And let me put it this way: the physician decides last but not least at some point or I decide against the physician's intention or the physician's main suggestion or first suggestion."
03	**Subjective item difficulty**			Participants were asked if answering this item was rather easy or rather hard for them.			
03.1		Subjective item difficulty item 1 (focusing the decision)					
03.1.E			Easy		8	8	
03.1.H			Hard	Participants reported difficulties remembering the situation to be rated.	3	3	
03.2		Subjective item difficulty item 2 (sharing the decision)					
03.2.E			Easy		7	7	
03.2.H			Hard		4	4	
03.3		Subjective item difficulty item 3 (presenting options)					
03.3.E			Easy		9	9	

03.3.H		Hard		1	1
03.3.M		Missing		1	1
03.4	Subjective item difficulty item 4 (informing on options)				
03.4.E		Easy		10	10
03.4.U		Unclear	No statement regarding subjective item difficulty	1	1
03.5	Subjective item difficulty item 5 (supporting comprehension)				
03.5.E		Easy		11	11
03.6	Subjective item difficulty item 6 (eliciting preferences)				
03.6.E		Easy		9	9
03.6.N		Neutral		1	1
03.6.H		Hard	Participant reported it hard to answer this item as there was only one option.	1	1
03.7	Subjective item difficulty item 7 (deliberating the decision)				
03.7.E		Easy		7	7
03.7.H		Hard		3	3
03.7.U		Unclear		1	1
03.8	Subjective item difficulty item 8 (selecting an option)				
03.8.E		Easy		8	8
03.8.H		Hard		3	3
03.9	Subjective item difficulty item 9 (planning actions)				
03.9.E		Easy		8	8
03.9.H		Hard		2	2
03.9.M		Missing		1	1
